# Mechanical compatibility of sol–gel annealing with titanium for orthopaedic prostheses

**DOI:** 10.1007/s10856-015-5611-3

**Published:** 2015-12-21

**Authors:** Andrew I. M. Greer, Teoh S. Lim, Alistair S. Brydone, Nikolaj Gadegaard

**Affiliations:** School of Engineering, University of Glasgow, Glasgow, G12 8LT UK

## Abstract

Sol–gel processing is an attractive method for large-scale surface coating due to its facile and inexpensive preparation, even with the inclusion of precision nanotopographies. These are desirable traits for metal orthopaedic prostheses where ceramic coatings are known to be osteoinductive and the effects may be amplified through nanotexturing. However there are a few concerns associated with the application of sol–gel technology to orthopaedics. Primarily, the annealing stage required to transform the sol–gel into a ceramic may compromise the physical integrity of the underlying metal. Secondly, loose particles on medical implants can be carcinogenic and cause inflammation so the coating needs to be strongly bonded to the implant. These concerns are addressed in this paper. Titanium, the dominant material for orthopaedics at present, is examined before and after sol–gel processing for changes in hardness and flexural modulus. Wear resistance, bending and pull tests are also performed to evaluate the ceramic coating. The findings suggest that sol–gel coatings will be compatible with titanium implants for an optimum temperature of 500 °C.

**Electronic supplementary material:**

The online version of this article (doi:10.1007/s10856-015-5611-3) contains supplementary material, which is available to authorized users.

## Introduction

It is known that the native surface oxide present upon titanium metal is essential for inhibiting potentially toxic ion release from titanium [1], furthermore specific phases of crystallography have been shown to be beneficial for integrating titanium with bone [2]. Sol–gel technology has been established over several decades and previously suggested as a surface coating for orthopaedic implants [3]. One of the fundamental processing steps for sol–gel technology is the annealing stage; performed to transform the precursor solution into a ceramic layer. By controlling the annealing temperature one may control the crystallography of the resultant ceramic coating [4]. In addition to controlling crystallography, surface texture may also be varied. Nanopatterning is a widely reported mechanism for influencing stem cell behaviour [5–7]. Recently titania based sol–gels were combined with nanotopogarphies to heighten the osteoinductive response over planar titanium in in vitro studies [8]. Despite the extensive evidence supporting the application of sol–gel coatings to titanium metal implants from a biological perspective, there has been no evaluation to date confirming the mechanical compatibility.

Literature suggests that the required temperature to convert sol–gel solution into ceramic lies within the range of 300–700 °C [9–11]. Literature also suggests that sintering titanium based orthopaedic implants may be detrimental for the longevity of the implant, as it is at increased chance of mechanical shear failure [12]. Therefore it is crucial to examine and document whether the application of such a sol–gel layer, in particular the annealing stage, induces any mechanical or structural side effects for the bulk metal substrate which may compromise the longevity of the device.

This study focuses on the mechanical and structural characterisation of titanium, the prevalent material for orthopaedics at present, before and after application of a titanium-based sol–gel. Grade II commercially pure titanium (cpTi (II)) substrates were tested pre- and post-coating at distinct annealing temperatures for variation in hardness and flexural modulus. The ceramic coating itself is also of acute interest from a structural perspective so bend and pull testing were performed to evaluate the coating integration strength. This property is of paramount importance for medical implants as loose particles can induce inflammation and trigger cancers [13–15]. To evaluate the composition and morphology of the ceramic materials produced following heat treatment at various temperatures, X-ray photoelectron spectroscopy (XPS) and Raman spectroscopy were deployed.

Due to the facile and versatile nature of sol–gel chemical synthesis [13], the precursor may be readily tailored to include different elements should the coating require specific properties such as increased wear resistance. However this study is focused primarily on the effect of annealing, as such in order to evaluate this variable thoroughly only one composition of sol–gel (titanium-based) is documented in the main manuscript. Additional data for sol–gel-derived alumina and zirconia coatings (popular materials for arthroprosthetics [16]) may be found in Supplementary Section S5.

## Methods

### Sol–gel synthesis

All chemicals were sourced from Sigma-Aldrich. The solution is prepared by mixing 0.96 ml of diethanolamine (99 %) with 5.54 ml of 1-hexanol (99 %) and 0.10 ml of deionised water. Diethanolamine is a solid at room temperature so the source bottle requires heating above the melting point of 28 °C before a decantation may be made. The mixture should be vigorously stirred for 10 min before slowly adding 3.40 g of the desired metalalkoxide (titanium butoxide) while stirring. Stirring should continue in a sealed vial for 2 h to ensure complete dissolution of the chemicals. The typical shelf life of this sol–gel precursor is 1 month when used frequently in an environment with ~30 % humidity.

### Thermal gravimetric analysis

10 ml of sol–gel was subjected to a continuous ramp thermal gravimetric analysis from room temperature (21 °C) to 700 °C on a TA Instruments TGA Q500. The resultant percentile weight and change in weight were recorded dynamically against temperature. This analysis indicates several chemical characteristics including the relevant temperatures for the removal of solvents and the ultimate temperature required to remove the majority of carbon to leave a stable ceramic material. The change in weight (first order derivative) is plotted because it may indicate if there are two mass-loss reactions overlapping or occurring consecutively.

### Raman analysis

As previously mentioned, the structural phase of the ceramic may be controlled through the annealing process. Titanium dioxide is known to exist in several phases which produce distinctive electromagnetic spectra under Raman microscopy. Thus the titanium-based sol–gel was annealed at a range of temperatures and examined using Raman microscopy. Polished cpTi (II) surfaces were spin coated with the titanium sol–gel and annealed to: 300, 500 and 700 °C. Raman analysis was performed on a Renishaw InVia Raman microscope in order to identify the structural phase of the annealed coatings and compare them to an untreated metal control. The 514 nm wavelength laser source was deployed, and the Renishaw CCD sensor was utilised at five exposures per second with accumulations of 10 traces per plot to reduce noise.

### XPS set-up

A SAGE 100 system (Specs GmbH, Germany) was used as for the XPS analysis. Base pressure in the analysis chamber was approximately 2e-7 mbar. The X-ray source was MgKα operated at an anode voltage of 12.5 kV and 250 W of power. Spectra were recorded, following a 50 min Ar sputter, at a take-off angle of 90°. The pass energy for the hemispherical analyzer was 50 eV for these survey scans. Spectra were analyzed using casaXPS software, and the elemental composition was determined by integration of peak areas using a standard Shirley background.

### Abrasion testing

It is highly desirable for any medical implant that the coating has strong integrity and is able to resist abrasion without delaminating. An abrasion test was set-up with P320 grit SiC paper placed upon the face of cpTi (II) samples with a pressure of 900 Pa and dragged 20 mm across the surface via a line and pulley featuring a hanging mass of 50 g. The samples were then examined via optical microscopy to evaluate the level of wear in terms of total scratch length (mm). Sol–gel coated samples were annealed at 300, 500 and 700 °C and compared to polished cpTi controls.

### Vickers microhardness testing

In order to determine whether or not the sol–gel annealing process has any impact on the mechanical integrity of the underlying titanium metal both Vickers microhardness testing and 3-point bend testing were carried out. For the Vickers microhardness testing a Wilson Wolpert Model 401MVA microhardness tester was used to probe through the surface oxide and into bulk material. For this experiment 1.25 mm thick cpTi (II) sheets were used. For these experiments the machine was fitted with a square pyramid diamond tip. A 0.01 kg load was applied to the tip. Six iterations were carried out for each sample. Optical microscopy was used to view and manually measure the diagonals of the indent. A value of Vickers hardness for each tested substrate was calculated automatically by the tool (once it was provided with the length of the diagonals on the observed imprint).

### Three-point bend testing

For the three-point bend testing a Zwick Z250 materials testing device was used. The machine can withstand the test load up to a maximum force of 250 kN and has a positioning resolution up to ±2 μm. A round forcing tip of radius 10 mm was used and the distance between supports was set to 60 mm. The titanium samples were 77.0 × 29.0 × 1.1 mm in size. The test speed was set to 10 mm/min. The force required to bend the titanium and the induced deflection were measured at a rate of 10 Hz. A Veho VMS-004 Delux USB microscope was attached to record video footage of the sample during the testing.

### Pull testing

Pull tests were carried out using a Zwick Z250 materials testing machine upon a cpTi control and cpTi samples featuring the titanium-based sol–gel coating after annealing at the various maximum temperature levels. Permabond ET500 17 two-component structural fast setting engineering epoxy adhesive was used to glue the machine mounts onto the specimen surfaces. The adhesive is specified as having a maximum shear strength of 14 MPa tested against a mild-steel surface at room temperature. The samples were examined by optical microscopy following the pull testing and both the tensile force being applied and the deflection of the tool was recorded during the tests. Annotations and illustrative photographs may be found in the Supplementary Section S3.

## Results and discussion

### Thermal gravimetric analysis

**Fig. 1:**
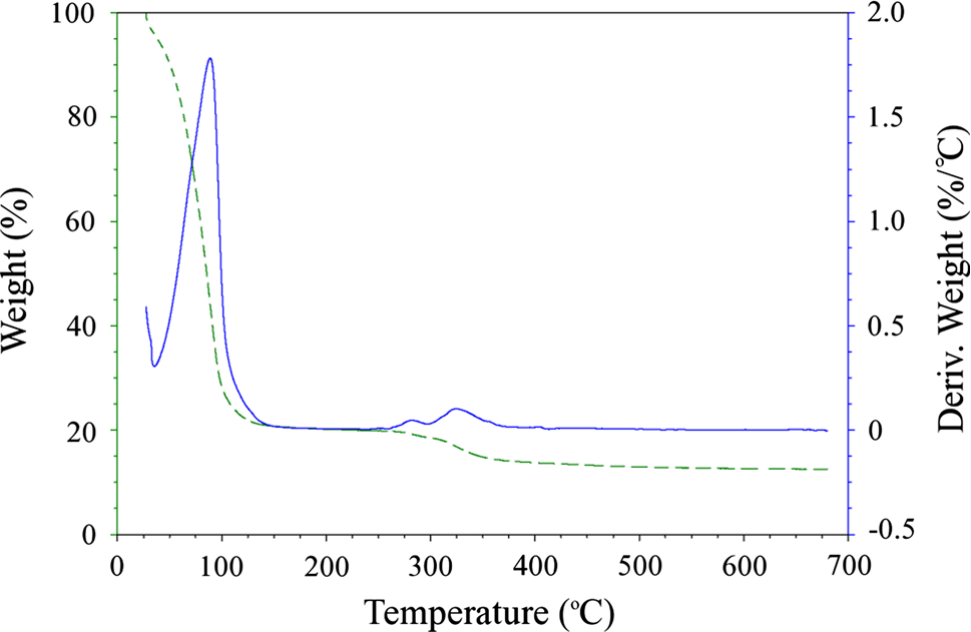
Thermal gravimetric analysis for the reported titanium-based sol–gel chemistry. The *dashed line* represents percentile weight loss (*left hand axis*) and the *solid line* represents the first-order derivative for the percentile weight (*right hand axis*). Both against temperature from room temperature to 700 °C

TGA was performed upon the synthesised sol–gel in order to evaluate the temperature range applicable for evaporating and pyrolytically decomposing the novel chemical composition. The resolved spectrum of mass against heating temperature may be observed in Fig. 1. Analysis of the graph shows that there is a steady, large drop off in mass until 120 °C which corresponds with solvent evaporation. A carbon-containing titania-based coating is present until 350 °C. Further heating (tested to 700 °C) appears to release the remaining organic components from the material to realise a thermally stable ceramic layer.

### Raman analysis

**Fig. 2:**
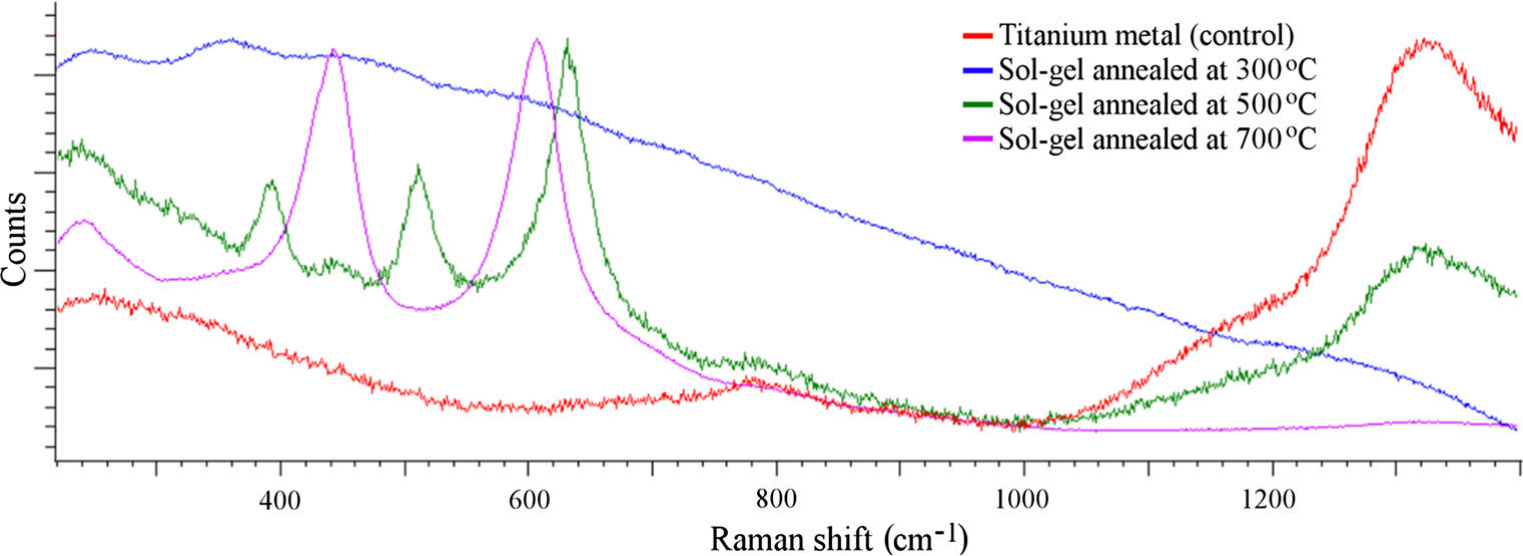
Raman spectra from an accumulation of ten traces per sample using a 785 nm wavelength laser in static mode on the Renishaw CCD sensor at five exposures per second for titanium featuring the presence of sol–gel coatings after being annealed at various temperatures including a control spectrum from titanium metal. The plot for the coating annealed at 300 °C shows a falling background signal due to the non-crystalline nature of the coating. The coating annealed at 500 °C features the characteristic anatase spectrum and the 700 °C coating features the characteristic rutile spectrum [17] (Color figure online)

Raman analysis indicated that crystal morphology can indeed be tailored through selection of the maximum sintering level. Titania has several distinct phases and is thus a good example of phase control. Figure 2 displays that amorphous, anatase and rutile titania can be achieved by sintering the sol–gel at 300, 500 and 700 °C respectively. The reason the sample coated with sol–gel and annealed at 300 °C failed to provide a strong spectrum is because the film still contains a significant level of carbon (as evident from TGA) and has not yet crystallised thus it is an impure, amorphous coating. As the annealing temperature was increased to 500 °C one may observe that the spectrum for anatase [17] was indeed detected because this temperature was sufficient to burn out the majority of the carbon species and induce crystallisation. By increasing the temperature further to 700 °C the titania underwent a further phase change and conformed to the expected rutile spectrum [17].

### XPS analysis

It was determined from TGA that sol–gel sintering stabilises in weight around 350 °C. XPS analysis was executed to quantify the level of carbon retained in the coatings annealed above 350 °C. Silicon was used as the substrate so as not to interfere with identification of the spectral peaks of the coating. The resultant XPS spectra may be found in the Supplementary Section S1. The derived percentile atomic composition of coatings annealed at 500 and 700 °C are shown in Table 1. It was discovered that carbon content is comparable to a cpTi (II) control after annealing at 500 °C and, despite the change in crystallography, there is no reduction in carbon content when the temperature is increased to 700 °C. Of more pertinence is the annealing ramp rate. Increasing the ramp rate from 2 to 10 °C/min inhibits carbon release.

**Table 1:** Percentile atomic composition for various annealing conditions

Surface	Ti	Ti	Ti	Ti
Coating	Native oxide	TiO_2_ precursor	TiO_2_ precursor	TiO_2_ precursor
Anneal T_max_	N/A	700 °C	500 °C	500 °C
Anneal rate	N/A	2 °C/min	2 °C/min	10 °C/min
Titanium level	25.0 %	30.8 %	31.0 %	25.0 %
Oxygen level	70.5 %	62.6 %	63.5 %	58.2 %
Carbon level	4.5 %	6.6 %	5.5 %	16.8 %

### Abrasion testing

An evaluation of how the maximum annealing temperature effects the wear resistance of the titanium based sol–gel coating is documented in Table 2. From inspection of Table 2 it can be seen that resistance to scratching improves as the annealing temperature is increased. From the previous tests it can be deduced that the abrasion results are subsequent of the structural phase change in the oxide coating from amorphous through anatase to rutile. This finding is as expected since rutile is known to be harder than amorphous or anatase oxides due to its dense crystalline structure.

**Table 2:** Summary of data for mechanical tests on cpTi (II) following sol–gel treatment and annealing at different temperatures, including an untreated cpTi (II) control

Mechanical test	Maximum annealing temperature			
	Untreated cpTi (II) (control)	300 °C	500 °C	700 °C
Total length of abrasive scaring (mm)	38.3 ± 7.8	34.7 ± 7.9	32.4 ± 4.8	16.2 ± 2.3
Micro hardness (HV0.01)	151.8 ± 6.9	152.1 ± 7.7	149.9 ± 5.8	455.8 ± 54.2
Maximum flexural modulus (MPa)	841.1 ± 18.8	854.3 ± 32.5	836.9 ± 37.9	912.4 ± 38.7
Pull stress at point of failure (MPa)	6.8 ± 0.8	10.2 ± 0.6	9.1 ± 3.0	7.0 ± 0.8

Standard deviation from three trials per test are displayed next to the average value for each sample in each test

### Vickers microhardness testing

Comparing the Vickers microhardness before and after sol–gel treatment at both 300 and 500 °C found no noticeable variation between the coated metal substrates. These results are also displayed in Table 2. As can be seen, from inspection of Table 2, the Vickers microhardness remains on average 150 HV (1471 MPa) for all test conditions up to 500 °C. After annealing at 700 °C the hardness of the cpTi (II) metal increased three fold to 456 HV (4472 MPa). Hardness tests were repeated with and without the sol–gel coating and the hardness increase remained constant. There is no known phase change which occurs in pure titanium metal at 700 °C, but the surface of the metal may form a hard rutile-like oxide coating naturally at this temperature without the presence of a sol–gel layer. Hardness tests were also evaluated at 100 times greater load (1 kg) so as to achieve larger indents which may be more accurately measured. The results mirrored the 0.01 kg runs. Optical micrographs of the 1 kg indents may be found in Supplementary Section S2. There it may be observed that stress cracks have propagated from the corners of the indent on both the sol–gel coated and untreated samples that were annealed at 700 °C. This indicates that the cpTi (II) is more brittle after heat treatment at 700 °C regardless of sol–gel presence, but is not affected by heating to 500 °C which correlates to the hardness results.

### Three-point bend testing

**Fig. 3:**
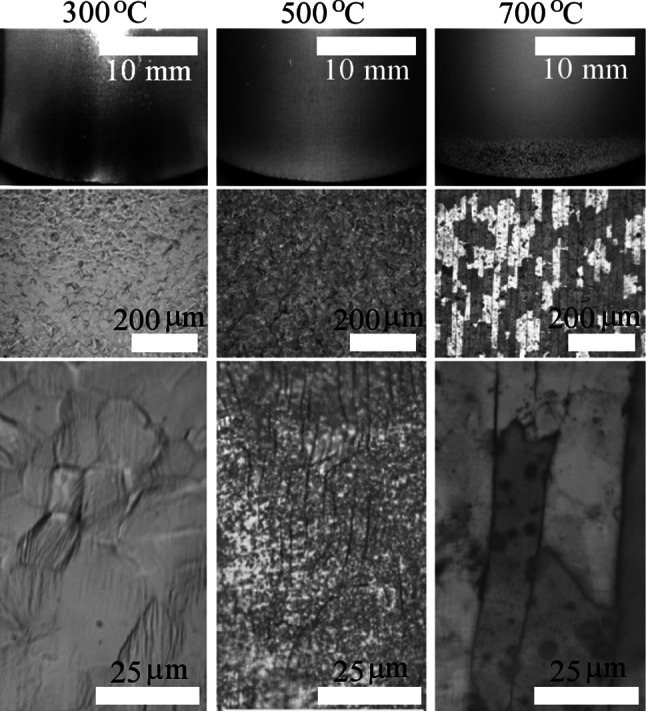
Optical image analysis of bend testing at 30 mm deflection for sol–gel coated cpTi (II) samples annealed at various maximum temperature (denoted at the *top* of each *column*)

In connection with the hardness tests, bend testing was also deployed to evaluate how the flexural modulus was affected by maximum annealing temperature. The flexural modulus followed the trend of previous mechanical tests with only the 700 °C data point noticeably different in magnitude. Values for the flexural modulus are displayed in Table 2. Optical micrographs of the sintered coatings at the point of maximum deflection (30 mm) are shown in Fig. 3. From these images it is clear that the ductility of the surface oxide decreases with increased maximum annealing temperature as the 300 °C sample exhibits no stress cracks, the 500 °C sample features nanoscale cracks and the 700 °C sample features a mosaic of 30 by 100 µm shards running normal to the direction of curvature.

### Pull testing

In addition to evaluating the scratch resistance of the coatings, a pull test was also evaluated to determine the integration strength of the thin film ceramic layer. For the 500 and 700 °C annealed sol–gel samples either the glue split (cohesive failure) or the glue to ceramic bond failed (adhesive failure). There was faint discolouration observed on the samples annealed at 300 °C. This is suspected to be delaminating of carbon species from the surface as it is known from TGA and Raman analysis that not all of the carbon present in the sol–gel is burnt off at this temperature. Pictorial analysis of these findings may be found in the Supplementary Section S3. The sol–gel coated samples required similar pressure to the cpTi (II) control before the system failed which indicates that the integration between the substrate and coating is robust and durable. Table 2 displays the pressure at the point of failure for each sample and the standard deviation from three trials.

## Discussion

This paper aimed to address whether the annealing stage, which is fundamental for sol–gel ceramic formation, has any detrimental impact upon the mechanical integrity of the underlying cpTi (II). An annealing furnace was ramped at 2 °C/min with variable maximum temperature: 300, 500 and 700 °C. It was discovered that 300 °C is not high enough to release all of the carbon from the coating. It is also known that there is no phase change in cpTi at this level so there was no mechanical compromise for the metal but equally there was no appreciable benefit as the semi-organic coating appeared more prone to delaminating than the alternatives. However, XPS analysis indicated that carbon content was comparable to titanium controls after annealing to 500 °C and, through Raman analysis, it was determined that the oxide is now of crystalline anatase phase. The findings of the mechanical testing suggest that application of titanium based sol–gel to titanium metal does not compromise the hardness or bending strength of cpTi (II) at temperatures up to 500 °C. In addition the integration strength between cpTi (II) and the titania coating annealed at 500 °C was sound with the epoxy or epoxy/ceramic interface failing every time at stress levels around 10 MPa. However increasing the annealing temperature further to 700 °C resulted in the cpTi (II) hardness increasing three fold and the plastic region bending strength increasing by 8.5 %. In correlation with these increases, the material was found to be more brittle after annealing at 700 °C. Stress cracks appeared in these samples during hardness testing, independent of the presence of a sol–gel coating.

## Conclusion

These results suggest that sol–gel treatment and annealing to 500 °C with a ramp rate of 2 °C/min does not compromise the mechanical attributes of titanium and an almost pure anatase surface may be realised at this temperature which has strong integration with the underlying metal. These results indicate that sol–gel technology should be non-problematic for load-bearing, cpTi (II) orthopaedic or dental devices providing annealing is performed at the apparent optimum 500 °C. The only complication of annealing at 500 °C is that the coating becomes less ductile, but this should not be an issue for orthopaedics or dentistry as such implants are pre-shaped before applying the sol–gel surface treatment.

## Electronic supplementary material

Supplementary material 1 (DOCX 5955 kb)
