# Supplementary material for: Mechanical compatibility of sol–gel annealing with titanium for orthopaedic prostheses
Source: J Mater Sci Mater Med. 2015 Dec 21;27:21. doi: 10.1007/s10856-015-5611-3 (PMC4686541; doi:10.1007/s10856-015-5611-3)
Supplement: Supplementary file 1 — Supplementary material 1 (DOCX 5955 kb) [file 10856_2015_5611_MOESM1_ESM.docx]

Mechanical compatibility of sol-gel annealing with titanium for orthopaedic prostheses

*Andrew I. M. Greer*, Teoh S. Lim, Alistair S. Brydone and Nikolaj Gadegaard*

School of Engineering, University of Glasgow, G12 8LT, UK
*E-mail: Andrew.Greer@glasgow.ac.uk

Supplementary

S*1. XPS plots:*

*
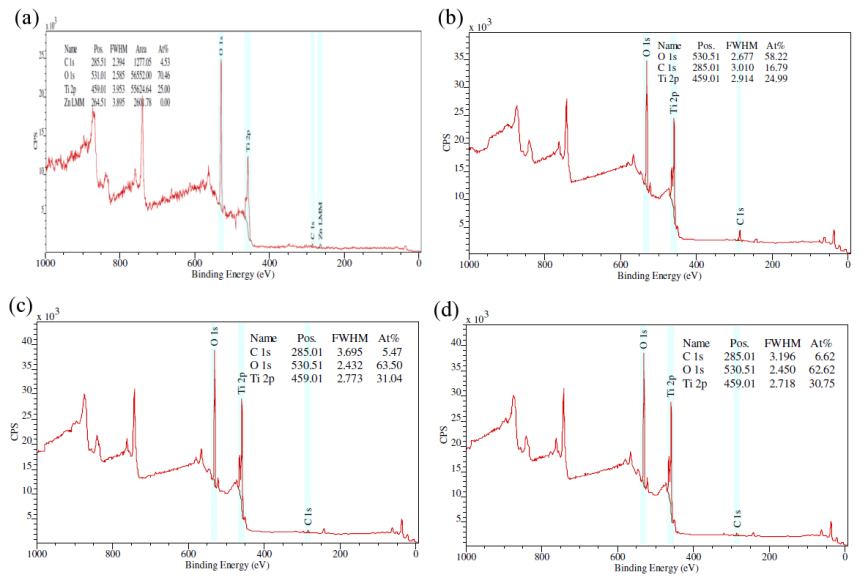
*

*Figure A1. XPS spectra for the three different ceramic layers produced by the three variants of sol-gel: (a) Al-based, (b) Zr-based and (c) Ti-based.*

*S2. Vickers hardness testing:*


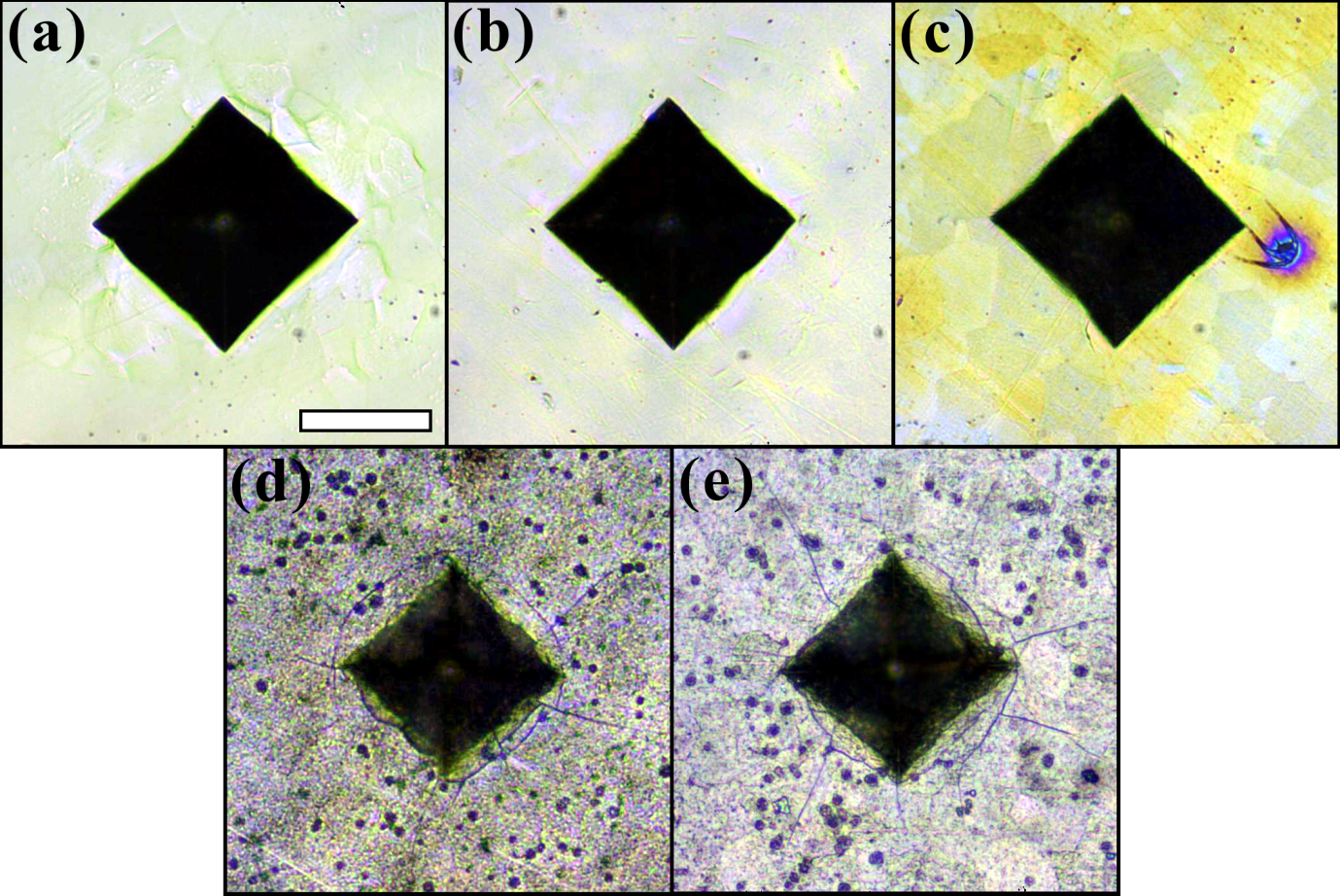


*Figure A2. Top-down optical micrograph of a square pyramidal Vickers indent at 1 kg load into samples of polished titanium metal with: (a) no coating (pre-anneal), (b) titanium sol-gel coating having been annealed at 300 °C, (c) titanium sol-gel coating having been annealed at 500 °C, (d) titanium sol-gel coating having been annealed at 700 °C, (e) no coating having been annealed at 700 °C. All images are at the same magnification and a 50 µm scale bar is shown in part (a) .*

*S3. Pull testing:*

**

*Figure A3. Schematic of pull test set-up.*

**

*Figure A4. Annotated photograph of pull test set-up.*

*
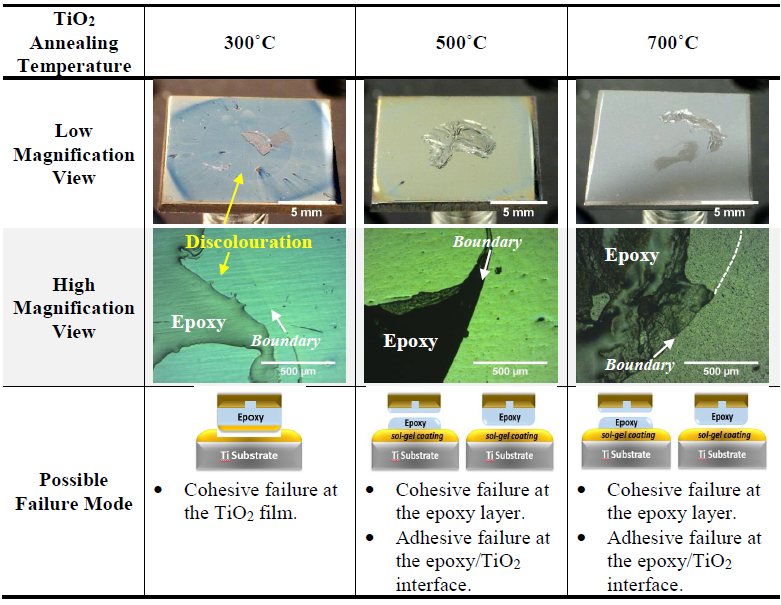
*

*Figure A5. Pull test pictorial analysis.*

*S4. Optical Metallurgy:*

*
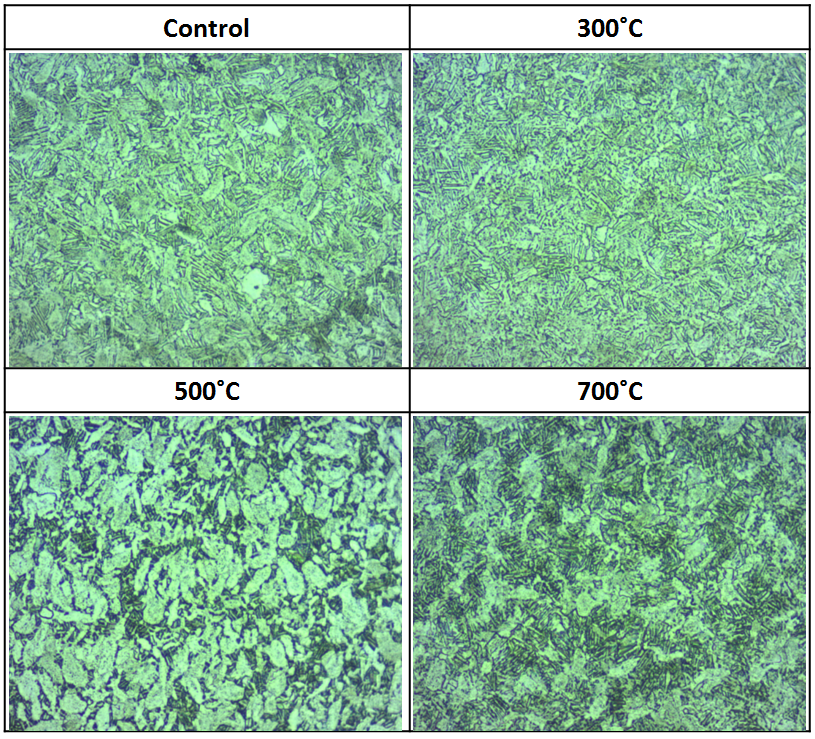
*

*Figure A6. Optical micrographs depicting the centre region from a polished and HF acid dipped cross-section face from a cpTi (II) rod after annealing at the labelled maximum temperature (control not annealed). All images at the same magnification, scale bar = 0.1 mm.*

*
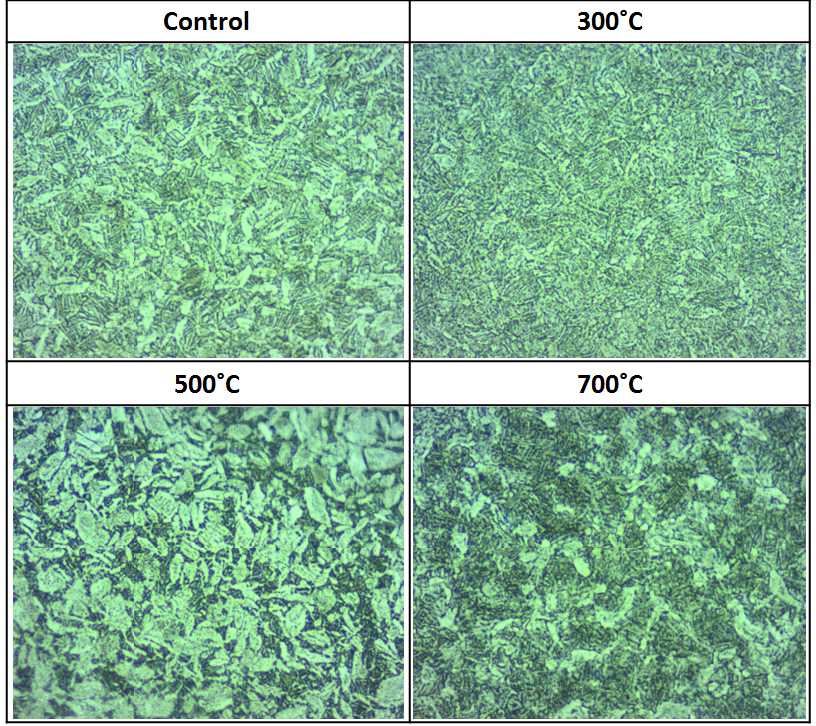
*

*Figure A7. Optical micrographs depicting the outer edge region from a polished and HF acid dipped cross-section face from a cpTi (II) rod after annealing at the labelled maximum temperature (control not annealed). All images at the same magnification, scale bar = 0.1 mm.*

S5. Alternative sol-gel compositions:

aluminium tri sec butoxide and zirconium butoxide replaced titanium butoxide in the documented recipe to change the composition of the formed ceramic. Figure A8 displays the TGA for the alternative compositions. After annealing the alternative sol-gels at 2 °C/min to 500 °C XPS spectra (Figure A9) were recorded as before following 50 min argon sputtering.

*
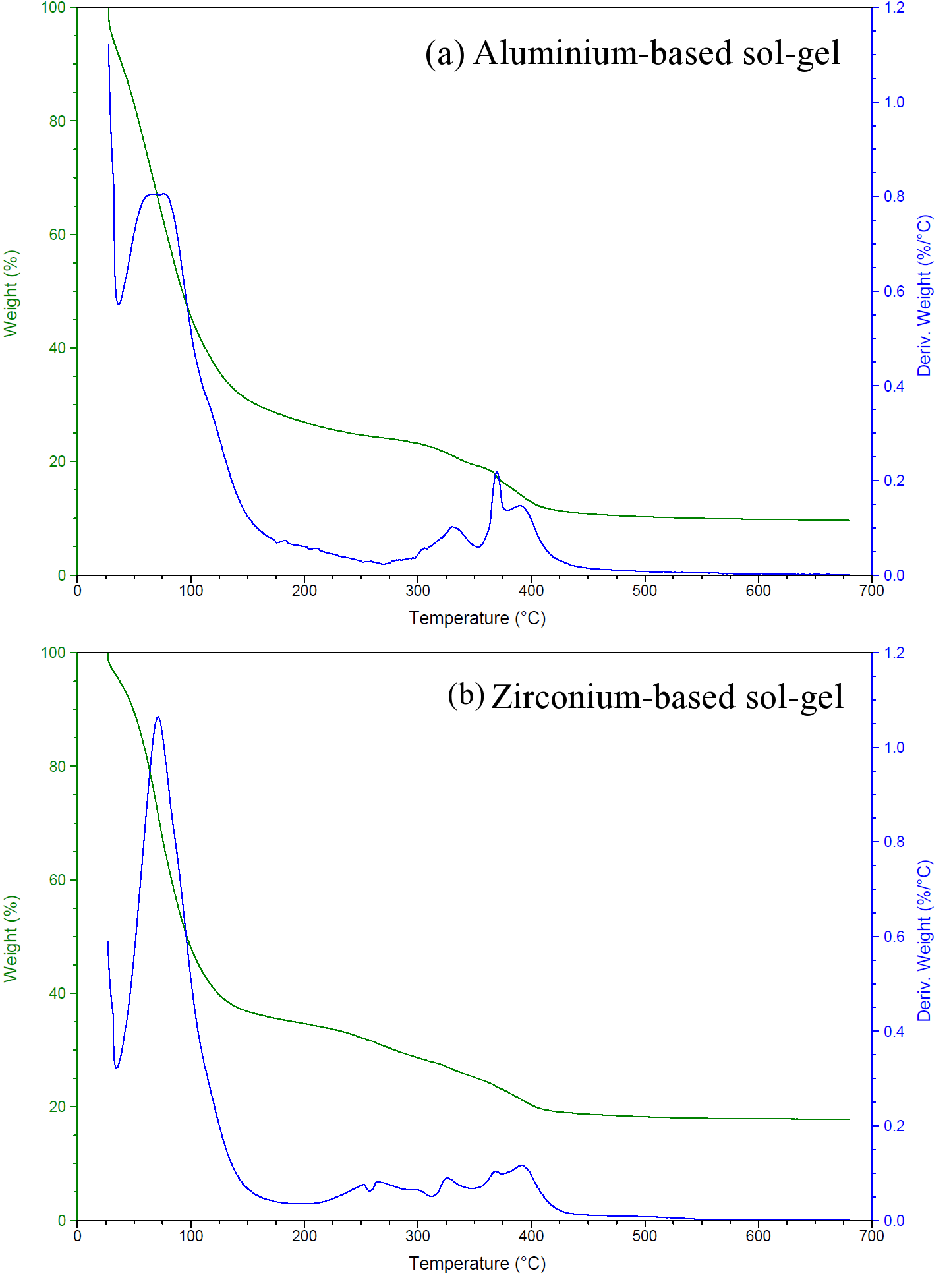
*

*Figure A8. Thermal gravimetric analysis for (a) Aluminium and (b) Zirconium based sol-gels synthesised using the same sol-gel recipe where the green line represents percentile weight loss (left hand axis) and the blue line represents first-order derivative of percentile weight (right hand axis) against temperature from room temperature to 700 °C.*

*
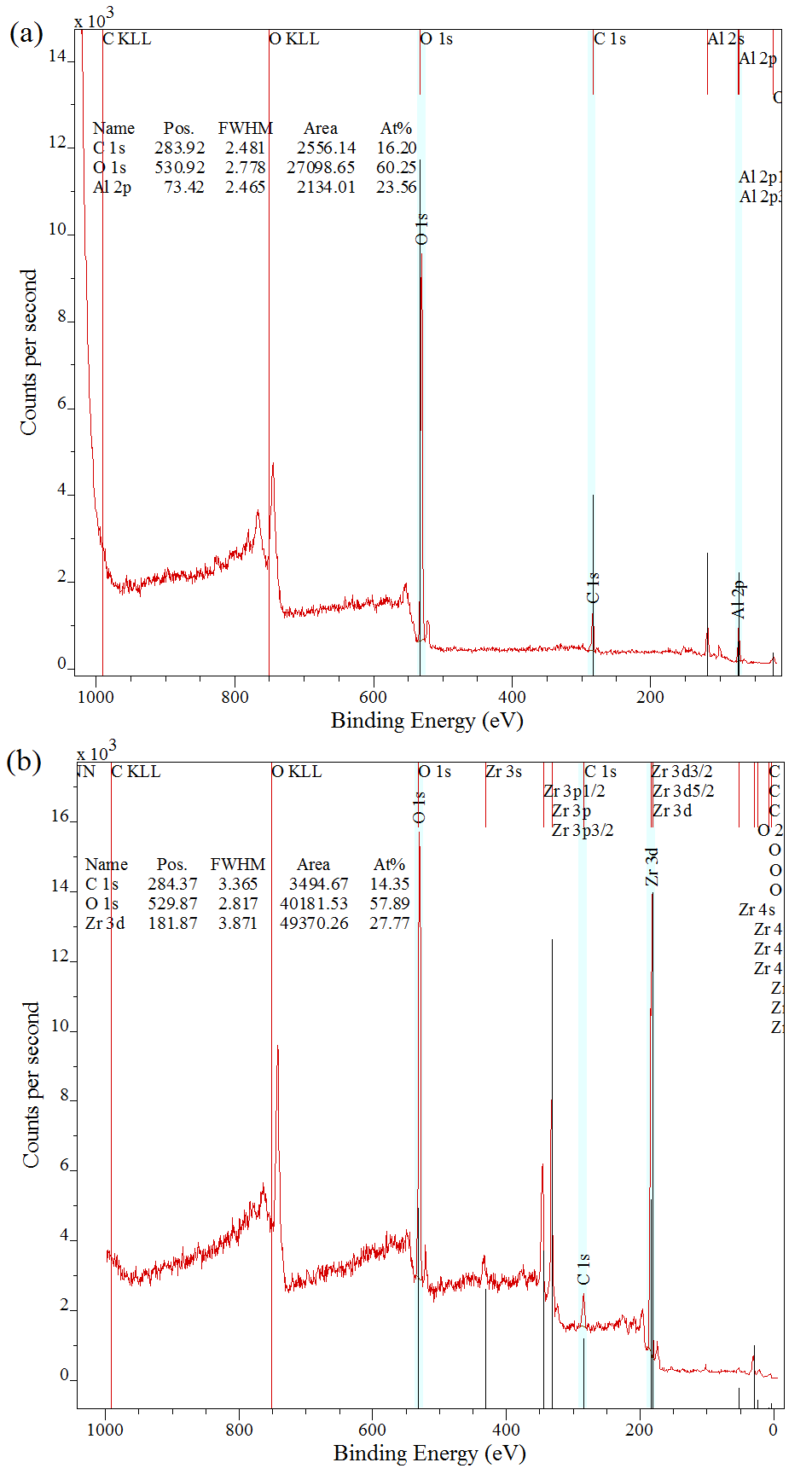
*

*Figure A9. XPS spectra for (a) Aluminium and (b) Zirconium based sol-gels* after annealing at 2 °C/min to 500 °C (following 50 min argon sputtering).

Being able to readily alter the elemental composition and phase of the ceramic coatings are useful aspects of the technology. As previously discussed alumina and zirconia coatings have already found popularity as highly stable, low wearing interfaces for arthroprostheses and are thus very relevant materials to synthesis and evaluate the abrasion resistance of. Figure A10 shows how the sol-gel composition can impact on resistance to wear with the sapphire (alumina) layer suffering less than half the wear induced in the titania counterpart at 500 °C.


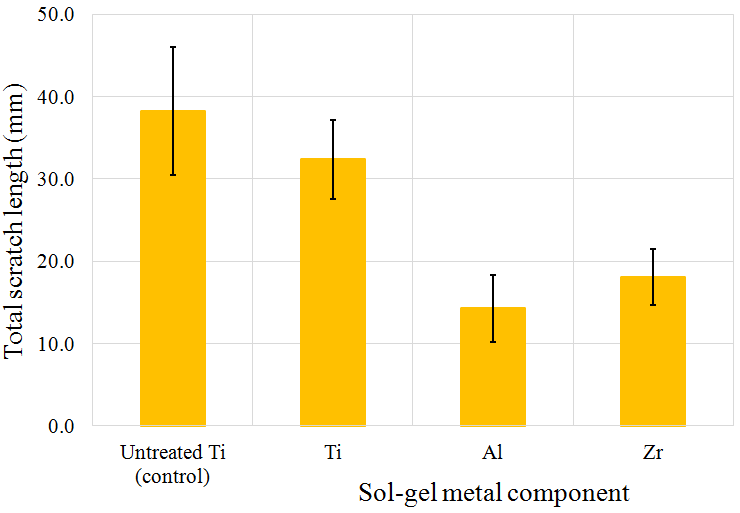


*Figure A10. Graph of abrasion resistance as measured by total scratch length per sample for titanium metal coated with each of the synthesised sol-gels tested after annealing at 500 °C with reference to an untreated titanium metal control. Error bars represent standard deviation from 3 specimens per sample.*
